# Infrastructure, service delivery, and staffing gaps in primary health care facilities in Lagos State, Nigeria: a state-wide assessment

**DOI:** 10.1186/s12982-025-01321-y

**Published:** 2025-12-31

**Authors:** Faith Oniyire, Tomike Ajose, Lateefah Andu, Abiola Adepase, Akin Abayomi, Olukemi Ogunyemi, Olusegun Ogboye, Olajumoke Oyenuga, Olajumoke Ibiyemi, Zainab Abdulrahman-Giwa, Adedapo Adejumo, Bukunyi Ajanaku, Babatunde Adewale, Hammed Mogaji

**Affiliations:** 1 Department of Healthcare Planning, Research and Statistics, Lagos State Ministry of Health, Lagos, Nigeria; 2 Research and Program Unit, Tropimanage Solutions Limited, Lagos, Nigeria; 3 Department of Public Health and Epidemiology, Nigerian Institute of Medical Research, Lagos, Nigeria; 4 Public Health Program, Department of Behavioral and Applied Social Sciences, Marian University, Indianapolis, Indiana USA

**Keywords:** Infrastructure quality, Service delivery, Healthcare staffing, Primary health care centers, Policy improvement

## Abstract

In this study, we conducted a state-wide assessment of infrastructure, service delivery and staffing to assess compliance with national recommended guidelines. We surveyed 279 (88%) of the 319 existing primary health care centers (PHCs) across the six health districts (HDs) in Lagos State using pretested electronic field forms. Data was analyzed using descriptive statistics, and chi-square test was used to ascertain significant differences in compliance across the HDs. Significance differences were established at *p* < 0.05. For infrastructure quality, 37% of the PHCs had access to service rooms, (range 31–50%, *p* = 0.05), 56% had functional toilets (range 48–72%, *p* = 0.07), 23% had staff accommodation (range 11–46%, *p* = 0.001) and 51% were over-burdened (range 18–68, *p* = 0.001). By service delivery, 65% had staff stations (range 50–75, *p* = 0.058), 59% had delivery rooms (range 43–78%, *p* = 0.009), 61% had maternity wards (range 48–86, *p* = 0.003), 53% had in-patient wards (range 40–62%, *p* = 0.14). By staffing, 48% had medical officers (range 33–57%, *p* = 0.16), 26% had nurse/midwives (range 20–34, *p* = 0.002), 29% had community health workers (range 18–36%, *p* = 0.011), 46% had health assistants (range 36–67%, *p* = 0.078), and 62% had laboratory technicians (range 44–75%, *p* = 0.12). Findings demonstrate critical gaps in infrastructure, service delivery, and staffing across Lagos State PHCs, and should guide investments in upgrading PHC facilities and strengthen workforce deployment in Lagos State.

## Introduction

Nigeria, with a population exceeding 227 million and an annual growth rate of 2.5%, continues to pursue a more responsive health care system [1, 2]. The country operates a three-tier system of government—federal, state, and local—comprising 37 states and 774 local government areas (LGAs) [3]. Health service delivery responsibilities are shared across these tiers. The Federal Government provides overall policy direction, planning, and technical support, coordinates state-level implementation of the National Health Policy and maintains health management information systems [3]. State governments manage secondary health facilities (general hospitals), some tertiary hospitals, and, in certain cases, primary health care centers. Local governments are responsible for operating primary health care facilities, which deliver basic medical services, community health, and sanitation programs [3].

Primary health care (PHC) represents the foundation of an effective health system and plays a pivotal role in achieving equitable health outcomes and strengthening population health [4–6]. PHCs are typically established in rural and underserved areas, where social determinants of health are most pronounced and where people live and work [4]. At this level, health services are adapted to meet local needs, cultural contexts, and community realities. Despite its recognized importance, PHC remains underfunded and undervalued in many countries, including Nigeria [4]. Strengthening PHC requires sustained investment, policy commitment, and adequate resource allocation to enhance its functionality and accessibility [7, 8]. Consequently, systematic assessments that identify programmatic and implementation gaps are critical for improving the quality-of-service delivery across PHCs and informing ongoing efforts aimed at optimizing health systems management.

Lagos is one of the most populous states in Nigeria, with an estimated population exceeding 20 million—approximately 10% of the nation’s total [9]. As the country’s primary economic hub, Lagos attracts substantial internal migration from other regions [10], contributing to rapid population growth and urban density which stretches existing PHC. Consequently, the demand for healthcare services and the need for an efficient health system response are considerably higher in Lagos compared to many other states in Nigeria. To strengthen the responsiveness of PHC, six health districts were recently established to enhance decentralization, improve governance, and facilitate quicker decision-making. However, despite these reforms, there has been no comprehensive, state-wide assessment of PHC infrastructure, service readiness, or staffing to guide investments and policy actions. Existing reports are fragmented, outdated, or limited to small geographic areas. This lack of current evidence creates major gaps for policy direction, resource allocation, and performance monitoring. Therefore, this assessment was undertaken to provide updated, district-level baseline evidence on the quality of PHC infrastructure, service delivery and staffing to identify priority gaps requiring policy action and targeted investment.

As an essential first step, it is imperative to assess the conditions of the structural elements that constitute the foundation of service delivery within the newly established HDs [11]. These structural elements encompass the physical, technical, and organizational components that serve as prerequisites for effective healthcare delivery [12]. The quality of structures within which care is provided directly influences the processes of care, which in turn affect patient outcomes. Indicators for assessing the quality of these structural components often focus on the setting of care—such as the adequacy of facilities and equipment, staffing ratios, qualifications of health personnel, and the efficiency of administrative systems. Hence, this study assessed the current conditions of PHCs across the six health districts in Lagos State, with the specific objectives of evaluating infrastructure adequacy, examining service delivery readiness, and determining staffing levels in relation to national recommended guidelines [13].

## Methods

### Ethics approval and accordance

This study received ethical approval from the Health Research Ethics Committee at the Lagos State Ministry of Health (LSMOH/BMGF/2024/01), and the six Health Districts. Prior to data collection, we engaged key representatives at the Health Districts and PHCs in a stakeholder meeting where information about the study’s objectives, procedures, and target population were provided. Participation in the study was made entirely voluntary for each recruited PHCs, and there were opportunities for replacements in case of PHCs that refused, were abandoned or non-functional. Informed consent was obtained from the directorate of each PHC before commencement of interviews. All study procedures adhered to the ethical principles of the Declaration of Helsinki (1964; most recently amended in 2008) and relevant national guidelines governing research involving human participants.

### Study area

Lagos is the industrial hub and economic capital of Nigeria. It is in the southwestern part of Nigeria and bordered to the south by the Atlantic Ocean and the west by the Republic of Benin. Lagos has 6 HDs, 52 administrative areas known as Local Government Areas (LGAs) or Local Council Development Areas (LCDAs), 275 wards and 319 primary health care centers. The city of Lagos covers an immense area, with a total of 1,171.28 square kilometers (452.23 square miles), and harbors a debatable population of over 20 million people.

### Study design and selection of study sites

This study employed a cross-sectional design and was conducted in May 2024 across the six health districts (HDs) of Lagos State, which collectively host 319 primary health care centers (PHCs). The sampling unit was the individual PHC. To ensure representativeness across all wards and HDs, we developed a complete sampling frame of all 319 PHCs and arranged them based on geographic proximity within each ward. We then employed a stratified systematic sampling approach in which we selected one PHC randomly from each of the ward (the strata), and in wards with more than five PHCs, we sampled 1 PHC for every 5 PHCs, ensuring that wards with more PHCs contributed proportionally more while maintaining representation from all wards. PHCs that were found to be non-functional at the time of data collection were replaced with the next eligible PHC on the sampling list within the same ward. This process yielded a final sample of 279 PHCs, representing 88% of all PHCs in Lagos State. (Table 1)

**Table 1:** Overview of study locations/sampling sites

Health District (HD)	Name of Area	Number of LGA/LCDA	Number of Wards	Number of PHCs selected
HD1	Alimosho - Ojokoro	10	45	44
HD2	Kosofe - Ikorodu	11	45	45
HD3	Epe – Eti-Osa East	9	53	50
HD4	Apapa - Surulere	4	39	42
HD5	Ajeromi - Oriade	10	48	51
HD6	Ikeja - Ejigbo	8	45	47
Total		52	275	279

*HD* Health District,* LGA* Local Government Area,* LCDA* Local Development Council Area,* PHCs* Primary Health Centers

### Selection of respondents within phcs

At each PHC, we conducted an initial site visit to identify a focal respondent who possessed sufficient institutional knowledge of the facility’s operations. Eligible respondents included the facility head (PHC Director) or a designated senior staff member such as the Officer-in-Charge, senior nurse, or most experienced health worker on duty. These individuals were selected purposively because they were the staff members most knowledgeable about infrastructure, service delivery processes, resource availability, and daily operational challenges of the PHC.

Only one respondent was interviewed per facility to ensure consistency and avoid duplication of operational perspectives. Questionnaire administration occurred on a scheduled return visit to minimize workflow disruption and allow respondents adequate time to prepare relevant records when necessary. In addition to the structured interview, direct observations of infrastructure, equipment, infection-prevention resources, and service-delivery indicators were completed using a standardized checklist.

### Data collection

Pretested closed-ended questionnaires and field forms adapted from the national guidelines for recommended conditions of PHC establishment in the country [16], were used to document the conditions of infrastructure, service delivery station and staffing profile across each of the selected PHCs. Two types of questionnaires were administered via Android or iOS devices: (i) a PHC facility form and, (ii) a structural quality questionnaire. Questions related to infrastructure targeted presence/absence of adequate service rooms, functional toilets, staff accommodation, kitchen, population served, amongst others. Also, questionnaires on service delivery targeted presence/absence of adequate staff stations, delivery rooms, maternity wards and in-patient wards amongst others. For staffing profile, questions were sufficiency of key personnel such as medical officers, nurse/midwives, community health workers, health assistants and laboratory technicians (Table 2). All data were collected electronically and securely transferred to a remote backup server following each interview.

### Data analysis

The analysis and interpretation of data were guided by the National Guidelines for Primary Health Care Infrastructure and Minimum Service Package in Nigeria [16]. For infrastructure and service delivery, compliance was assessed based on the presence (coded as “Yes”) or absence (coded as “No”) of required components, whereas staffing compliance was evaluated by comparing the number of available staff in each cadre against the nationally recommended thresholds and categorized as lacking, not satisfactory (below recommendation), satisfactory (meets recommendation), or above recommended. All indicators had complete data except for “population served”, which was missing for 9 PHCs. Descriptive analyses—including frequencies and proportions—were conducted in R software (v.4.3.3), with results aggregated at the health district level. Chi-square test was used to test for significant differences in compliance across the six health districts, with statistical significance set at *p* < 0.05.

**Table 2:** List of main indicators for infrastructure, service delivery stations and personnel and the minimum requirements based on National guidelines for primary health care infrastructure and minimum service package in Nigeria

SN	Infrastructure		Service Delivery		Staffing	
	Indicator	Minimum recommendation	Indicator	Minimum recommendation	Indicator	Minimum recommendation
1	Population served	1 PHC should serve 10,000–20,000 persons	Waiting/reception area	Required, but number not specified in guideline	Medical officer	1 staff
2	Rooms	At least 13 rooms should be available	Staff Station*	Required, but number not specified in guideline	Community Health Officer	1 staff
3	Secured building (fenced, netted windows, doors)	Required, but number not specified in guideline	Pharmacy dispensing unity	Required, but number not specified in guideline	Nurse/Midwife	4 staff
4	Potable water source	Required, but number not specified in guideline	Delivery room	Two units should be available	Community Health Workers	3 staff
5	Functional toilets	Required, but number not specified in guideline	Maternity lying in section	Required, but number not specified in guideline	Pharmacy technician	1 staff
6	Waste disposal site	Required, but number not specified in guideline	In-patient ward section	Required, but number not specified in guideline	Junior Community Health worker	6 staff
7	Sanitary water collection point	Required, but number not specified in guideline	Laboratory	Required, but number not specified in guideline	Environmental officer	1 staff
8	Staff accommodation	2 units of 1 bed-room flat should be available	Medical records area	Required, but number not specified in guideline	Medical Records Officer	1 staff
9	Electricity (national grid)	Required, but number not specified in guideline	Injection dressing area	Required, but number not specified in guideline	Laboratory technician	1 staff
10	Electricity (alternative sources)	Required, but number not specified in guideline	Store	Required, but number not specified in guideline	Health Attendant/Assistant	2 staff
11	Kitchen	Required, but number not specified in guideline	–	–	Security personnel	2 staff
12	–	–	–	–	General maintenance staff	1 staff

*Staff Station is typically a central operational point where clinical and administrative staff carry out essential coordination tasks

## Results

A total of 279 (88%) of the 319 existing PHCs were surveyed across 238 wards, with an average of 47 (42–51) PHCs per health district.

### Compliance level for infrastructure quality

Across the 279 PHCs assessed, infrastructure readiness varied considerably, with significant inter-district differences for several indicators. Overall security was relatively strong, with 77% of PHCs classified as secured (*p* = 0.264). Access to potable water was high (77%) but showed sharp disparities across districts (*p* = 0.001), ranging from 66% in HD3 to 95% in HD1. Waste disposal sites were widely available (84%; *p* = 0.28) with minimal variation. Sanitary water collection points were present in 72% of PHCs, though marked differences were observed (*p* < 0.001), from 48% in HD3 to 84% in HD2. Electricity access from the national grid was similarly high (77%; *p* = 0.001) but varied substantially as only 50% of HD3 facilities were connected compared to 89% in HD2. Alternative energy sources were available in 75% of PHCs (*p* = 0.007), ranging from 64% in HD3 to 84% in HD2.

Several critical gaps were identified. Only 14% of PHCs served the recommended population size (10,000–20,000), while most were either overstretched (51%) or underserved (35%). Structural adequacy was limited, with just 37% of facilities meeting the recommended number of rooms (*p* = 0.503). Functional toilets were available in 56% of PHCs, with notable differences between districts (48% in HD1 vs. 72% in HD6). Staff accommodation was available in only 23% of locations and varied widely (*p* < 0.001), from 11% in HD6 to 46% in HD3. Kitchen availability was also low (25%), with large inter-district disparities (*p* = 0.001) ranging from 13% in HD2 to 53% in HD5.

### Compliance level for service delivery stations

Most facilities (90%) had a waiting or reception area, though coverage ranged from 82% in HD3 to 98% in HD1 and HD5 (*p* = 0.035). Pharmacy dispensing units were widely available (85%), with proportions spanning from 76% in HD3 to 94% in HD6 (*p* = 0.174). Laboratory services were present in 72% of PHCs, ranging from 60% in HD3 to 84% in HD1 (*p* = 0.112). Medical records areas were available in 77% of PHCs but showed significant inter-district variation—54% in HD3 compared to 89% in HD6 (*p* < 0.001). Injection/dressing areas (82%) and storage rooms (74%) demonstrated relatively uniform coverage with minimal district differences (*p* > 0.05).

In contrast, several critical service-delivery gaps were identified. Staff stations were available in only 65% of PHCs, with district coverage ranging from 53% in HD2 to 75% in HD5 (*p* = 0.058). Delivery rooms were present in 59% of facilities, with marked disparities—from 43% in HD4 to 78% in HD5 (*p* = 0.009). Similarly, maternity lying-in sections were available in just 61% of PHCs, varying significantly from 48% in HD4 to 86% in HD5 (*p* = 0.003). In-patient wards were present in 53% of PHCs, with a district range of 40% in HD4 to 62% in HD6 (*p* = 0.143), indicating moderate inadequacy in admission capacity across the state.

### Compliance level for staffing

Across the 279 PHCs, staffing adequacy varied widely. Community Health Officers were among the better-staffed categories, with 75% of facilities meeting recommended levels, although district differences were significant, ranging from 60% in HD3 to 93% in HD1 (*p* = 0.018). Similarly, 86% of PHCs had pharmacy technicians with district proportions ranging from 72% in HD5 to 98% in HD2 (*p* = 0.010). There were also more medical records officers in 71% of PHCs, with district-level coverage ranging from 52% in HD1 to 84% in HD2 (*p* = 0.082). Laboratory technicians were also present in adequate number in 62% of PHCs but ranged from 44% in HD4 to 75% in HD1 (*p* = 0.122).

However, significant staffing gaps persisted across many essential cadres. Medical officers were present in only 48% of PHCs, ranging from 33% in HD2 to 58% in HD4 (*p* = 0.163). Nurse/midwife staffing were present in only 26% of the PHCs, ranging from 20% in HD2 to 33% in HD5 (*p* = 0.002). Junior Community Health Extension Workers (JCHEWs) had the largest deficits, with only 2% of PHCs meeting the benchmark (*p* = 0.122). Health assistants were also insufficient, with only 46% of the PHCs meeting the requirements, ranging from 36% in HD6 to 67% in HD5 (*p* = 0.078). Environmental health officers were short staffed, with 45% of facilities above the recommended staffing level, though substantial district variation was observed (*p* = 0.001), from 20% in HD3 to 62% in HD1. Security personnel showed wide variation (*p* = 0.001), with only 44% of facilities adequately staffed, ranging from 31% in HD2 to 70% in HD1 (*p* = 0.001). General maintenance staff were also present in only 28% of the PHCs (*p* = 0.100, Tables 3, 4 and 5).

**Table 3:** Compliance level for infrastructure quality

Indicators	*N* = 279	HD 1	HD 2	HD 3	HD 4	HD 5	HD 6	*p*-value
		44	45	50	42	51	47	
Population served*								**0.001**
Appropriate (10,000-20000)	38 (14)	2 (4.7)	5 (11)	14 (28)	4 (10)	8 (16)	5 (11)	
Overserved (> 20000)	137 (51)	25 (58)	30 (68)	9 (18)	21 (54)	30 (61)	22 (49)	
Underserved (< 10000)	95 (35)	16 (37)	9 (21)	27 (54)	14 (36)	11 (23)	18 (40)	
Sufficient rooms								0.503
No	175 (63)	22 (50)	31 (69)	33 (66)	27 (64)	31 (61)	31 (66)	
Yes	104 (37)	22 (50)	14 (31)	17 (34)	15 (36)	20 (39)	16 (34)	
Secured building (fenced, windows, doors)								0.264
No	65 (23)	11 (25)	8 (18)	18 (36)	9 (21)	9 (18)	10 (21)	
Yes	214 (77)	33 (75)	37 (82)	32 (64)	33 (79)	42 (82)	37 (79)	
Availability of potable water source								**0.001**
No	63 (23)	2 (4.5)	10 (22)	17 (34)	15 (36)	16 (31)	3 (6.4)	
Yes	216 (77)	42 (95)	35 (78)	33 (66)	27 (64)	35 (69)	44 (94)	
Availability of functional toilets								0.071
No	123 (44)	23 (52)	23 (51)	25 (50)	21 (50)	18 (35)	13 (28)	
Yes	156 (56)	21 (48)	22 (49)	25 (50)	21 (50)	33 (65)	34 (72)	
Availability of waste disposal site								0.283
No	46 (16)	5 (11)	10 (22)	12 (24)	8 (19)	5 (9.8)	6 (13)	
Yes	233 (84)	39 (89)	35 (78)	38 (76)	34 (81)	46 (90)	41 (87)	
Availability of sanitary water collection point								**0.001**
No	79 (28)	8 (18)	7 (16)	26 (52)	15 (36)	13 (25)	10 (21)	
Yes	200 (72)	36 (82)	38 (84)	24 (48)	27 (64)	38 (75)	37 (79)	
Availability of Staff accommodation								**0.001**
No	214 (77)	34 (77)	38 (84)	27 (54)	34 (81)	39 (76)	42 (89)	
Yes	65 (23)	10 (23)	7 (16)	23 (46)	8 (19)	12 (24)	5 (11)	
Access to electricity (national grid)								**0.001**
No	63 (23)	8 (18)	5 (11)	25 (50)	7 (17)	11 (22)	7 (15)	
Yes	216 (77)	36 (82)	40 (89)	25 (50)	35 (83)	40 (78)	40 (85)	
Access to electricity (alternative sources)								**0.007**
No	70 (25)	11 (25)	7 (16)	18 (36)	9 (21)	17 (33)	8 (17)	
Yes	209 (75.2)	33 (75)	38 (84.4)	32 (64)	33 (79)	34 (67)	39 (83)	
Availability of Kitchen								**0.001**
No	208 (75)	36 (82)	39 (87)	35 (70)	35 (83)	24 (47)	39 (83)	
Yes	71 (25)	8 (18)	6 (13)	15 (30)	7 (17)	27 (53)	8 (17)	

*p*-value in bold shows significant differences

*HD* Health District; *missing records for 9 PHCs; records for2 (n = 1); HD4 (n = 3), HD5 (n = 2); HD6 (n = 2); ** The numbers in the parenthesis are %

**Table 4:** Compliance level for service delivery stations

Indicators	*N* = 279	HD 1	HD 2	HD 3	HD 4	HD 5	HD 6	*p*-value
		44	45	50	42	51	47	
Availability of waiting/reception area								**0.035**
No	27 (10)	1 (2)	6 (13)	9 (18)	6 (14)	1 (2)	4 (9)	
Yes	252 (90)	43 (98)	39 (87)	41 (82)	36 (86)	50 (98)	43 (91)	
Availability of Staff Station *								0.058
No	99 (35)	15 (34)	21 (47)	17 (34)	21 (50)	13 (25)	12 (26)	
Yes	180 (65)	29 (66)	24 (53)	33 (66)	21 (50)	38 (75)	35 (74)	
Availability of pharmacy dispensing unity								0.174
No	41 (15)	4 (9)	6 (13)	12 (24)	7 (17)	9 (18)	3 (6)	
Yes	238 (85)	40 (91)	39 (87)	38 (76)	35 (83)	42 (82)	44 (94)	
Availability of delivery room								**0.009**
No	115 (41)	16 (36)	17 (38)	24 (48)	24 (57)	11 (22)	23 (49)	
Yes	164 (59)	28 (64)	28 (62)	26 (52)	18 (43)	40 (78)	24 (51)	
Availability of maternity lying in section								**0.003**
No	109 (39)	18 (41)	19 (42)	22 (44)	22 (52)	7 (14)	21 (45)	
Yes	170 (61)	26 (59)	26 (58)	28 (56)	20 (48)	44 (86)	26 (55)	
Availability of in-patient ward section								0.143
No	132 (47)	17 (39)	26 (58)	25 (50)	25 (60)	21 (41)	18 (38)	
Yes	147 (53)	27 (61)	19 (42)	25 (50)	17 (40)	30 (59)	29 (62)	
Availability of laboratory								0.112
No	79 (28)	7 (16)	9 (20)	20 (40)	14 (33)	16 (31)	13 (28)	
Yes	200 (72)	37 (84)	36 (80)	30 (60)	28 (67)	35 (69)	34 (72)	
Availability of medical records area								**0.001**
No	63 (23)	7 (16)	9 (20)	23 (46)	7 (17)	12 (24)	5 (11)	
Yes	216 (77)	37 (84)	36 (80)	27 (54)	35 (83)	39 (76)	42 (89)	
Availability of injection dressing area								0.596
No	49 (18)	7 (16)	10 (22)	12 (24)	6 (14)	6 (12)	8 (17)	
Yes	230 (82)	37 (84)	35 (78)	38 (76)	36 (86)	45 (88)	39 (83)	
Availability of store								0.963
No	72 (26)	13 (30)	11 (24)	14 (28)	11 (26)	11 (22)	12 (26)	
Yes	207 (74)	31 (70)	34 (76)	36 (72)	31 (74)	40 (78)	35 (74)	

*p*-value in bold shows significant differences

*HD* Health District; *missing records for 9 PHCs; records for 2 (n = 1); HD4 (n = 3), HD5 (n = 2); HD6 (n = 2); ** The numbers in the parenthesis are %

**Table 5:** Compliance level for staffing

Indicators	*N* = 279	HD 1	HD 2	HD 3	HD 4	HD 5	HD 6	*p*-value
		44	45	50	42	51	47	
Number of medical officers (*n* = 1)								0.163
Above recommended	79 (28)	17 (39)	13 (29)	12 (24)	12 (29)	14 (27)	11 (23)	
Lacking	144 (52)	19 (43)	30 (67)	28 (56)	18 (42)	26 (51)	23 (49)	
Satisfactory	56 (20)	8 (18)	2 (4)	10 (20)	12 (29)	11 (22)	13 (28)	
Number of community health officers (*n* = 1)								**0.018**
Above recommended	99 (35)	21 (48)	12 (27)	14 (28)	17 (40)	15 (30)	20 (43)	
Lacking	69 (25)	3 (7)	14 (31)	20 (40)	7 (17)	17 (33)	8 (17)	
Satisfactory	111 (40)	20 (45)	19 (42)	16 (32)	18 (43)	19 (37)	19 (40)	
Number of nurse/midwives (*n* = 4)								**0.002**
Above r ecommended	59 (21)	9 (20)	9 (20)	10 (20)	8 (19)	12 (23)	11 (23)	
Lacking	30 (11)	3 (6.8)	2 (4)	14 (28)	7 (17)	4 (8)	0 (0)	
Not Satisfactory	176 (63)	27 (61)	34 (76)	25 (50)	26 (62)	30 (59)	34 (72)	
Satisfactory	14 (5)	5 (11)	0 (0)	1 (2)	1 (2)	5 (10)	2 (4.3)	
Number of pharmacy technicians (*n* = 1)								**0.010**
Above recommended	89 (32)	20 (45)	11 (24)	14 (28)	12 (29)	16 (31)	16 (34)	
Lacking	40 (14)	4 (10)	4 (9)	11 (22)	6 (14)	14 (28)	1 (2)	
Satisfactory	150 (54)	20 (45)	30 (67)	25 (50)	24 (57)	21 (41)	30 (64)	
Number of CHEWs (*n* = 3)								**0.011**
Above recommended	46 (16)	13 (30)	9 (20)	1 (23)	4 (10)	11 (22)	8 (17)	
Lacking	22 (8)	2 (4)	2 (4)	2 (4)	9 (21)	4 (8)	3 (6)	
Not Satisfactory	175 (63)	26 (59)	27 (60)	39 (78)	23 (55)	31 (60)	29 (62)	
Satisfactory	36 (13)	3 (7)	7 (16)	8 (16)	6 (14)	5 (10)	7 (15)	
Number of JCHEWs (*n* = 6)								0.122
Above recommended	2 (1)	0 (0)	1 (2)	0 (0)	0 (0)	0 (0)	1 (2)	
Lacking	216 (77)	39 (89)	38 (85)	42 (84)	28 (67)	39 (76)	30 (64)	
Not Satisfactory	60 (21)	5 (11)	6 (13)	8 (16)	14 (33)	12 (24)	15 (32)	
Satisfactory	1 (1)	0 (0)	0 (0)	0 (0)	0 (0)	0 (0)	1 (2)	
Number of environmental officers (*n* = 1)								**0.001**
Above recommended	104 (37)	25 (57)	18 (40)	8 (16)	11 (26)	15 (29)	27 (58)	
Lacking	152 (55)	17 (39)	19 (42)	40 (80)	26 (62)	32 (63)	18 (38)	
Satisfactory	23 (8)	2 (4)	8 (18)	2 (4)	5 (12)	4 (8)	2 (4)	
Number of health assistants (*n* = 2)								0.078
Above recommended	75 (27)	8 (18)	12 (27)	10 (20)	11 (26)	24 (47)	10 (21)	
Lacking	47 (17)	11 (25)	9 (20)	7 (14)	8 (19)	3 (6)	9 (19)	
Not Satisfactory	103 (37)	14 (32)	14 (31)	25 (50)	15 (36)	14 (27)	21 (45)	
Satisfactory	54 (19)	11 (25)	10 (22)	8 (16)	8 (19)	10 (20)	7 (15)	
Number of medical records officers (*n* = 1)								0.082
Above recommended	83 (30)	18 (41)	12 (27)	10 (20)	13 (31)	15 (29)	15 (32)	
Lacking	81 (29)	11 (25)	7 (16)	23 (46)	10 (24)	16 (32)	14 (30)	
Satisfactory	115 (41)	15 (34)	26 (57)	17 (34)	19 (45)	20 (39)	18 (38)	
Number of laboratory technicians (*n* = 1)								0.122
Above recommended	79 (28)	14 (32)	14 (31)	9 (18)	12 (29)	15 (29)	15 (32)	
Lacking	105 (38)	11 (25)	12 (27)	28 (56)	16 (38)	23 (45)	15 (32)	
Satisfactory	95 (34)	19 (43)	19 (42)	13 (26)	14 (33)	13 (26)	17 (36)	
Number of securities personnel (*n* = 2)								**0.001**
Above recommended	53 (19)	13 (29)	6 (13)	2 (4)	13 (31)	14 (27)	5 (10)	
Lacking	72 (26)	6 (14)	18 (40)	12 (24)	16 (38)	7 (14)	13 (28)	
Not Satisfactory	85 (30)	7 (16)	13 (29)	22 (44)	11 (26)	16 (32)	16 (34)	
Satisfactory	69 (25)	18 (41)	8 (18)	14 (28)	2 (5)	14 (27)	13 (28)	
Number of general maintenances staff (*n* = 1)								0.100
Above recommended	48 (17)	8 (18)	5 (11)	10 (20)	5 (12)	12 (23)	8 (17)	
Lacking	202 (72)	32 (73)	39 (87)	38 (76)	32 (76)	31 (61)	30 (64)	
Satisfactory	29 (11)	4 (9)	1 (2)	2 (4)	5 (12)	8 (16)	9 (19)	

*p*-value in bold shows significant differences

*HD* Health District; *missing records for 9 PHCs; HD1 (n = 1), HD2(n = 1); HD4 (n = 3), HD5 (n = 2); HD6 (n = 2).*Number in parentheses for each indicator is the recommended threshold based on [16]; ** The numbers in the parenthesis are %

## Discussion

Providing high-quality PHC depends on well-equipped, adequately staffed, and properly resourced health facilities with the necessary infrastructure, essential medicines, and consumables. Nigeria’s Ward Minimum Health Care Package illustrates a minimum healthcare package defined by several nations [14]. As determined by the National Primary Health Care Development Agency, this package comprises a set of health interventions provided in PHCCs daily at little or no cost to clients [15] and supported by a government financial mechanism [15]. The package contains government-defined minimum criteria for PHC center personnel resources, equipment, medication, infrastructure, and services [16]. Hence, in this study we have described the quality of these structural components to guide investments and policy direction [13].

On infrastructure, we have found significant structural deficiencies across the PHCs, that are likely to impact on the quality and accessibility of healthcare in Lagos State. Foremost the PHCs serve large and densely populated areas, lack the infrastructure, personnel, and essential resources necessary to meet healthcare needs effectively. Nearly half of the PHCs are overstretched, serving more than 20,0000 residents within their catchment areas, indicating substantial gaps in healthcare access across health districts. Many facilities suffer from inadequate space for consultation and treatment rooms, which contributes to overcrowding, reduces patient satisfaction, and increases the risk of infections [17]. Additionally, equitable access to safe and potable water and sanitation for all, has been promoted through the Sustainable Development Goal (SDG) 6 [18] and the global WASH in Health Care Facilities [19]. Our findings on water and sanitation infrastructure directly relate to these targets, and evidently sanitation remains a critical challenge, as only about half of the PHCs have functional toilets, while others lack consistent access to potable water, which undermines basic hygiene and infection control practices, and could be a reason for poor patronage [20]. These findings are in consonance with those reported locally from Enugu [8], and in a recent systematic review, with notable deficits across rural areas [21]. The current CDC estimates also put about 50% of healthcare facilities to be without access to piped water, 33% without improved sanitation, and 39% without soap for handwashing [20]. Challenges in water quality, sanitation and waste management therefore demand comprehensive, multi-sectoral approaches for improvement [21, 22].

For service delivery, a significant proportion of the health facilities do not have adequate maternal and inpatient rooms, and this could likely restrict access to safe maternal care, a vital component of healthcare given the high burden of maternal health challenges. The SDG 3.1 aims to reduce the global maternal mortality ratio to fewer than 70 deaths per 100,000 live births by 2030 [23]. In Nigeria, the goal is to reduce maternal mortality ratio (MMR) below 140 per 100,000 live births by 2030 [24]. Achieving this target would be hardly possible unless quality maternal health care service is implemented in all health care settings. Poor infrastructure such as lack of water, sanitation, electricity, and scarcity of functional delivery and inpatient wards would limit the PHCs’ ability to manage patients needing extended care beyond outpatient services, thereby escalating referrals to higher-level facilities and placing additional strain on the healthcare system [25]. This service and infrastructure shortfall would directly affect the ability of PHCs to deliver comprehensive and effective healthcare, particularly to vulnerable populations in underserved areas.

Staffing presents another critical barrier to effective healthcare delivery. The shortage of healthcare workers in rural and remote areas remains a growing concern in both developing and developed countries [26]. In Sub-Saharan Africa, there is a more serious human resource crisis in the health sector, with the number of healthcare workers grossly insufficient to achieve population health goals [27]. The shortage of medical officers, nurses, and midwives is also pronounced, and could have direct impact on patient care quality and outcomes [27, 28]. Recent assessments from PHCs in Enugu also reported shortage of medical officers, pharmacy and laboratory technicians and nurses/midwives [8]. While community health workers (CHEWs) are relatively more available, Junior CHEWs remain significantly underrepresented, further widening the gap in accessible, community-based healthcare services. To enhance healthcare delivery, addressing this disparity in staffing is essential. Also, we found that operational support and maintenance services are also lacking in many PHCs. Only 23% of PHCs provide accommodation for healthcare personnel, and this could be a factor that contributes to high absenteeism and retention challenges [29, 30, 31], especially in rural and underserved locations. Security personnel are limited, which risks the safety of both staff and patients and could further discourage healthcare providers from working in remote areas. The availability of general maintenance staff is also inadequate, affecting facility upkeep and potentially leading to a decline in the quality of care over time. Power and water inconsistencies would disrupt routine operations and compromise service reliability, especially in facilities dependent on these utilities to store medicines and operate medical equipment. The interplay between poor infrastructure, limited-service delivery capacity, and severe staffing shortages likely reinforces the systemic weaknesses observed across PHCs. For example, the scarcity of staff accommodation and poor facility conditions might have deter qualified professionals from accepting or retaining rural postings. This is reflected in the critically low availability of key cadres, particularly nurse-midwives. Such shortages could directly impact maternal mortality, especially in settings where delivery rooms and lying-in wards were also not available. Addressing these structural barriers holistically is therefore essential for strengthening frontline service delivery in Lagos State.

While our study focused on the quality of the conditions within the existing PHCs, the strain on PHC is yet another understudied issue. There are also notions that Nigeria has more PHCs and reasonable geographic access when compared to other countries [32]. However, this doesn’t capture within heterogeneity, as the continuous increase of population, migration and urbanization rate have increased the demand for public health services [33]. In Nigeria, migration has traditionally been driven by economic opportunities and security challenges [10]. Lagos, situated in southwestern Nigeria, has long been a major destination for migrants and has evolved into the country’s economic hub and the largest metropolis in West Africa [10]. Lagos experiences the highest rate of human migration from neighboring states and countries compared with any other state in Nigeria [10], which places additional strain on PHCs that are already scarce, and not equitably shared across the rural and urban areas [34]. A more practicable action would require using recent census-updates to forecast needs and establishment of new PHCs in areas where populations are underserved.

Overall, our study has highlighted the need for an urgent intervention to ensure that the PHCs are available in sufficient numbers, and their conditions are adequate to allow provision of safe and reliable care. Specifically, the residents within the health districts would benefit from upgrade of PHC facilities and strengthening of workforce deployment and their retention. This study has some strengths and limitations. Foremost, we utilized more indicators from the national guidelines to benchmark PHC performance, providing a more comprehensive assessment of structural readiness across facilities. The sampling of 88% of all existing PHCs and inclusion of multiple districts also allows for cross-district comparison within Lagos’s diverse urban environment. However, a major limitation is that we did not capture process or outcome dimensions of quality, and we have focused our survey on public facilities, excluding the private ones. Also, we relied on self-reported information from PHC staff which could introduce reporting bias particularly for indicators based on administrative records rather than direct observation. Lastly, our findings cannot be generalized to other states in Nigeria with different health system structures, resource levels, or contextual challenges. These limitations should be considered when interpreting our results.

## Conclusion

Our findings highlight an urgent need for strategic investment in PHC infrastructure, staffing, and essential resources to improve service quality and equity across Lagos State. Scaling PHCs in proportion to population density, particularly in underserved districts, will be essential to reduce overcrowding and improve maternal and child health outcomes. Enhancing staffing through recruitment, retention incentives, and improved living conditions for healthcare personnel can stabilize service delivery. Ensuring consistent access to water, sanitation, power, and security will create safer and more functional care environments. Targeted policy reforms aligned with national PHC guidelines and the State’s UHC framework can transform Lagos PHCs into resilient, high-performing facilities capable of meeting the health needs of diverse and rapidly growing communities.

## Data Availability

The datasets are available upon reasonable request to the corresponding author.
